# Broad-Spectrum Antiviral Activity of Influenza A Defective Interfering Particles against Respiratory Syncytial, Yellow Fever, and Zika Virus Replication In Vitro

**DOI:** 10.3390/v15091872

**Published:** 2023-09-04

**Authors:** Lars Pelz, Elena Piagnani, Patrick Marsall, Nancy Wynserski, Marc Dominique Hein, Pavel Marichal-Gallardo, Sascha Young Kupke, Udo Reichl

**Affiliations:** 1 Bioprocess Engineering, Max Planck Institute for Dynamics of Complex Technical Systems, 39106 Magdeburg, Germany; 2 Bioprocess Engineering, Otto von Guericke University Magdeburg, 39106 Magdeburg, Germany

**Keywords:** respiratory syncytial virus, yellow fever virus, Zika virus, defective interfering particles, broad-spectrum antiviral

## Abstract

New broadly acting and readily available antiviral agents are needed to combat existing and emerging viruses. Defective interfering particles (DIPs) of influenza A virus (IAV) are regarded as promising options for the prevention and treatment of IAV infections. Interestingly, IAV DIPs also inhibit unrelated viral infections by stimulating antiviral innate immunity. Here, we tested the ability of IAV DIPs to suppress respiratory syncytial, yellow fever and Zika virus infections in vitro. In human lung (A549) cells, IAV DIP co-infection inhibited the replication and spread of all three viruses. In contrast, we observed no antiviral activity in Vero cells, which are deficient in the production of interferon (IFN), demonstrating its importance for the antiviral effect. Further, in A549 cells, we observed an enhanced type-I and type-III IFN response upon co-infection that appears to explain the antiviral potential of IAV DIPs. Finally, a lack of antiviral activity in the presence of the Janus kinase 1/2 (JAK1/2) inhibitor ruxolitinib was detected. This revealed a dependency of the antiviral activity on the JAK/signal transducers and activators of transcription (STAT) signaling pathway. Overall, this study supports the notion that IAV DIPs may be used as broad-spectrum antivirals to treat infections with a variety of IFN-sensitive viruses, particularly respiratory viruses.

## 1. Introduction

Viral infections pose a serious health burden. Respiratory syncytial virus (RSV) infections represent the second-most common cause of infant death [1] but can also result in considerable disease in older adults [2,3]. For prophylaxis, two vaccines based on the RSV prefusion F protein are recommended for elderly people [4], and one is recommended for infants [5]. However, only antivirals can be used to treat acute infections. For instance, the small-molecule drug ribavirin is applied for the treatment of severe RSV infections in high-risk immunocompromised infants [6]. However, ribavirin is associated with a low antiviral effect, high costs, possible toxicity in patients, and risks for health care workers [6,7,8,9,10]. Infections with flaviviruses are another concern as they can result in high morbidity and mortality. Furthermore, as vector-born RNA viruses that can emerge unexpectedly in human populations, they constitute a serious global health challenge. Most infections are caused by yellow fever virus (YFV), Zika virus (ZIKV), dengue virus (DENV), and West Nile virus, which are primarily transmitted via mosquitoes to humans [11]. Vaccines are only approved for YFV, DENV, and Japanese encephalitis virus. No small molecule antiviral agents have been approved to treat acute flavivirus infections so far [12]. In a mouse infection model, the antibiotic fidaxomicin [13], which binds and inhibits the ZIKV polymerase, was shown to suppress ZIKV propagation. In addition, it was demonstrated that the nucleoside analog ribavirin [14] could be of potential use for treatment of humans.

Interferons (IFNs) of type-I (e.g., IFN-α and -β) and type-III (e.g., IFN-λ) are considered an option to inhibit flavivirus and RSV infections [15,16]. For instance, the treatment with type-I and -III IFNs resulted in inhibition of ZIKV infections [17]. In addition, treatment with type-I IFN negatively correlated with disease severity for RSV [16], raising the potential for antiviral treatment. Nevertheless, recombinant type-I IFN therapies are costly [18] and pose the risk of adverse effects [19,20,21]. Therefore, the development of new antivirals with broad-spectrum activity which are safe, effective, and affordable is in high demand.

One such option is the use of defective interfering particles (DIPs) [22,23,24,25]. DIPs are naturally arising viral mutants that are found in a variety of RNA viruses [26,27,28,29,30,31,32,33,34,35,36,37], including influenza A viruses (IAV) [38,39,40,41,42,43,44,45,46,47,48]. Conventional IAV DIPs have a large internal deletion in one of their eight viral RNA (vRNA) segments, leading to a defect in virus replication. Furthermore, IAV DIPs inhibit IAV propagation in the context of a co-infection via “replication interference”. Here, the defective interfering (DI) vRNA is preferentially replicated relative to the full-length (FL), infectious standard virus (STV) counterpart. This leads to a depletion of cellular and viral resources and results in the inhibition of STV replication [47,49,50,51]. As a result, IAV DIPs suppress many strains, including seasonal, pandemic, and highly pathogenic avian IAV [39,41,42,52,53].

Next to replication interference, IAV DIP infection results in the induction of an antiviral state and suppresses not only the replication of IAV [42,54,55] but also the replication of unrelated viruses including severe acute respiratory syndrome coronavirus 2 (SARS-CoV-2) [22], pneumonia virus of mice [23], and influenza B virus [24]. In line with this, DIPs of DENV have been shown to confer an antiviral effect against ZIKV, YFV, RSV, SARS-CoV-2 [25], and various DENV subtypes [56], while DIPs of poliovirus inhibited SARS-CoV-2, Coxsackivirus B3, and IAV infections [33]. In this context, it was suggested for IAV DIPs that host-cell-derived pattern recognition receptors (PRRs) recognize pathogen-associated molecular patterns (PAMPs) such as FL and DI vRNAs [57,58]. This initiates a signaling cascade that activates IFN expression. Finally, IFN-stimulated genes (ISGs) are expressed, with some of the proteins conferring antiviral activity. These include myxovirus resistance protein 1 (Mx1), radical S-adenosyl methionine domain containing 2 (RSAD2), and IFN-induced transmembrane protein 1 (IFITM1).

To investigate whether IAV DIPs can also be used to treat other relevant viral infections, we studied their antiviral activity against the replication of the IFN-sensitive RSV, YFV, and ZIKV in co-infection experiments in vitro in human lung cells. For this, we used the IAV DIP “DI244” [39,59], a well-characterized DIP harboring a deletion in segment (Seg) 1. In addition, we tested “OP7”, a new type of IAV DIP that shows multiple point mutations in the vRNA of Seg 7 instead of an internal deletion [52]. DIP preparations were generated in cell cultures using laboratory-scale bioreactors [38,60]. We demonstrate that IAV DIPs inhibit RSV, YFV, and ZIKV propagation in vitro via stimulation of the innate antiviral immunity. Our results suggest that IAV DIPs might be a promising option for use as broad-spectrum antivirals to treat infections of many different IFN-sensitive viruses.

## 2. Materials and Methods

### 2.1. Cells and Viruses

Adherent human lung epithelial (A549, American Type Culture Collection (ATCC), #CCL-185) cells were maintained at 37 °C and 5% CO_2_ in Dulbecco’s Modified Eagle Medium (DMEM) supplemented with 10% fetal bovine serum (FBS, Merck, Darmstadt, Germany, #F7524). Adherent African green monkey kidney epithelial (Vero) cells (European Collection of Authenticated Cell Cultures (ECACC), #88020401) and porcine stable kidney (PS) cells (provided by M. Niedrig, Robert Koch Institute (RKI), Berlin, Germany) were routinely cultivated in Glasgow Minimum Essential Medium (GMEM) supplemented with 10% FBS and 1% peptone at 37 °C and 5% CO_2_. For YFV and ZIKV seed virus generation, Vero cells were cultivated in VP-SFM (Thermo Fisher Scientific, Waltham, MA, USA, #11681020) and GMEM (10% FBS, 1% peptone), respectively. Adherent HeLa-derived Epithelial Carcinoma (HEp-2) cells (ATCC, #CCL-23) were maintained in DMEM supplemented with 10% FBS, or without FBS (infection medium) for RSV seed virus production. Hep-2 cells were cultivated in RPMI1640 medium (Thermo Fisher Scientific, #21870084) supplemented with 2 mM L-glutamine and 10% FBS, or without FBS (infection medium) for 50% tissue culture infectious dose (TCID_50_) assay.

For infections, live attenuated YFV-17D (provided by M. Niedrig, RKI, Berlin, Germany) was used. ZIKV, originally isolated from whole blood specimens of ZIKV-positive adults in State of Espirito Santo, Brazil, was provided by Oswaldo Cruz Foundation, Brazil. Human RSV strain A2 (RSV A2) was obtained from ATCC (#VR-1540). OP7 and DI244 preparations (active and inactive, Table 1) were produced in a cell culture-based process, as described previously [38,60]; steric exclusion chromatography was used for purification [61,62].

### 2.2. Co-Infection Experiments

A549 and Vero cells were seeded at 0.5 × 10^6^ cells/well and 0.4 × 10^6^ cells/well, respectively, in 6-well plates and incubated for 24 h. Cells were washed prior to infection with 1× phosphate-buffered saline (PBS). Cells were infected with indicated viruses alone at a multiplicity of infection (MOI) of 10^−2^, or co-infected with 100 μL of active or inactive IAV DIPs (Table 1) at indicated dilutions. Cells were co-treated with ruxolitinib (Cayman Chemical, Ann Arbor, MI, USA, #11609), IFN-β-1a (PBL assay Science, Piscataway, NJ, USA, #11410), ribavirin (Cayman Chemical, #16757-5), and fidaxomicin (MedChemExpress, Monmouth Junction, NJ, USA, #HY-17580) at indicated concentrations. For RSV infection, additional pre-treatment (3 h) with ruxolitinib was conducted.

For ZIKV infections, wells were incubated with 2000 μL of serum- and sodium-bicarbonate-containing medium (A549: DMEM, 10% FBS, 1% sodium bicarbonate; Vero: GMEM, 10% FBS, 1% peptone, 5% sodium bicarbonate) containing the virus(es) and/or antivirals. YFV infections were conducted in 500 μL of serum-free medium, and incubation was performed for 4 h at 37 °C. Subsequently, YFV infected cells were washed with 1× PBS, and 2000 μL of corresponding serum-free medium was added. For YFV infections, pH was adjusted manually through the addition of 7.5% sodium bicarbonate to prevent pH from decreasing below 6.8. Supernatants were harvested at 48 h post-infection (hpi), and 72 hpi for YFV and ZIKV infections, respectively. After centrifugation at 2000× *g*, 5 min and 4 °C, cell-free supernatants were stored at −80 °C until virus quantification.

For RSV infections, wells were incubated with 250 μL of serum-free medium containing indicated virus(es) and antivirals and incubated for 2 h. Then, 1750 μL infection medium was added. Samples were taken at 72 hpi, followed by centrifugation at 300× *g*, 5 min, 4 °C. Next, cell-free supernatants were snap frozen in liquid nitrogen.

For intracellular RNA extraction, remaining cells were washed with 1× PBS and lysed with 350 µL of RA1 buffer (Macherey Nagel, Düren, Germany, #740961) containing 1% of β-mercaptoethanol. Purification of RNAs was conducted according to the manufacturers’ instructions. RNAs were stored at −80 °C.

### 2.3. Virus Quantification

For experiments with YFV and ZIKV, infectious virus titers were quantified via plaque assay as described recently [64] with few modifications. In brief, PS cells (0.2 × 10^5^ cells/well) were added to 24-well plates two days prior to infection. Cells were infected with 170 μL of serially diluted samples (each 1:10, two replicates per sample) and incubated for 4 h at 37 °C and 5% CO_2_. Subsequently, 600 μL of 1.6% carboxymethyl cellulose in GMEM (supplemented with 10% FBS and 1% peptone) was added to the virus-containing medium. Cells were then incubated (37 °C, 5% CO_2_) for 96 h (YFV) or 64 h (ZIKV). For fixation, 300 μL of glyoxal solution was used (15 min) and cells were stained with 300 μL of naphthalin black solution (30–60 min). Finally, plaques were counted and infectious virus titers expressed as plaque-forming units (PFU)/mL calculated based on the Spearman and Kärber method [65].

Quantification of infectious RSV titers were conducted by TCID_50_ assay in accordance to a previously published protocol [66] with few modifications. In brief, HEp-2 cells (0.2 × 10^5^ cells/well) were seeded to 96-well plates two days prior to infection. For infection, cells were washed with infection medium and 25 μL of serially diluted samples (each 1:5, eight replicates per sample) were added. After incubation (2 h, 37 °C, 5% CO_2_), 75 μL of infection medium was added to each well and cells were incubated for 96 h. Wells that showed the presence of syncytia and/or lytic cell death under the light microscope were scored positive for RSV. For detection of lytic cell death, cells were stained with 50 μL of crystal violet solution. The infectious virus titer was determined according to the Spearman and Kärber method.

### 2.4. Gene Expression Analysis

To measure mRNA expression levels, a real-time reverse transcription-quantitative PCR (RT-qPCR) method was utilized [22,52]. Purified RNA was reverse transcribed using Maxima H minus reverse transcriptase (Thermo Fisher Scientific, #EP0751) and an oligo (dT) primer according to the manufacturers’ instructions. Next, real-time qPCR was performed with gene-specific forward and reverse primers (Table 2) and 2× QuantiNova SYBR green PCR master mix (Qiagen, Hilden, Germany, #208056). The fold change in gene expression (relative to untreated, uninfected cells (mock infection control)) was calculated using the ΔΔCt method [67] using the reference housekeeping gene of glycerinaldehyd-3-phosphat-dehydrogenase (GAPDH).

### 2.5. Quantification of Intracellular IAV vRNAs

To quantify intracellular genomic vRNAs of IAV, a previously described real-time RT-qPCR method was used [22,52,60,71] with a different reagent for qPCR (2× QuantiNova SYBR green qPCR master mix, Qiagen, #208056). The method employs a primer system that allows gene-specific detection of individual IAV vRNAs [72]. RNA reference standards were used to facilitate absolute quantification.

## 3. Results

### 3.1. IFN-Dependent Inhibition of YFV Propagation by IAV DIP Co-Infection

**Figure 1:**
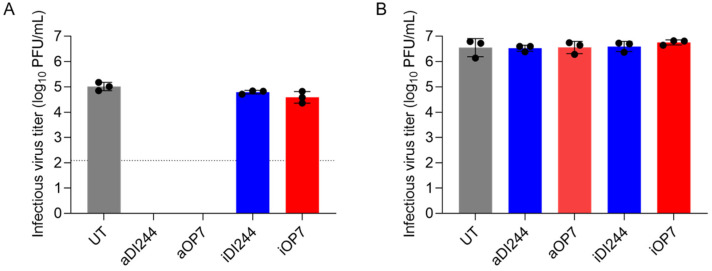
Inhibition of YFV replication and spread by IAV DIP co-infection in vitro in IFN-competent and -incompetent cells. Cells were infected with YFV alone at a MOI of 10^−2^ (UT, untreated) or co-infected with 100 μL of active (a) or inactive (i) IAV DIPs DI244 or OP7. Infections in IFN-competent A549 cells (**A**) or IFN-deficient Vero cells (**B**). The DIP material used was diluted to 1:20. Infectious virus release is indicated by the YFV plaque titer (PFU/mL) at 48 hpi. The figure depicts the results of three independent experiments. Dashed horizontal line shows the limit of detection (LOD). Error bars indicate the standard deviation (SD).

To study whether IAV DIPs suppress YFV propagation, we performed in vitro co-infection experiments in A549 (IFN-competent) and Vero cells (IFN-deficient [73,74,75,76,77,78]) (Figure 1). Cells were infected with YFV alone (untreated, UT) at a MOI of 10^−2^ or co-infected with purified and concentrated IAV DIPs (DI244 or OP7, diluted to 1:20) derived from cell culture-based production [38,60]. Co-treatment with active DIPs (aDI244 and aOP7, see Table 1) resulted in a strong inhibition of infectious YFV release in A549 cells, as no plaque titer was detected (Figure 1A). As expected, inactive DIPs (iDI244 and iOP7) conferred no apparent antiviral activity. Note that iDI244 and iOP7 were UV irradiated for 24 min, which results in an inactivation and degradation of DI vRNAs [38,60]. In Vero cells that are deficient in IFN production [74,76], co-infections with active and inactive DIPs did not display any antiviral activity (Figure 1B).

**Figure 2:**
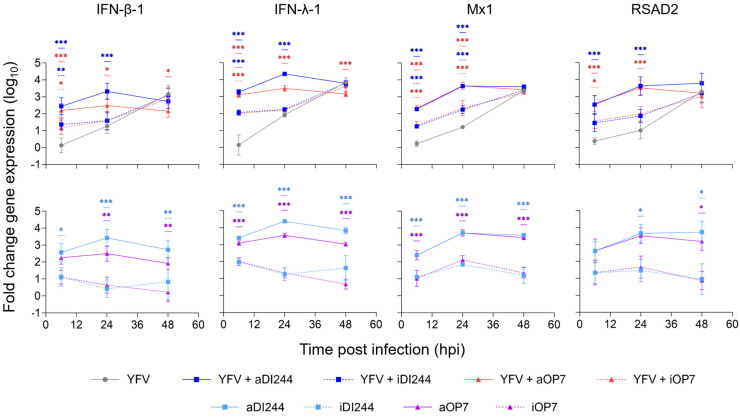
Induction of IFN-induced antiviral gene expression by IAV DIP infections. A549 cells were infected with YFV alone at a MOI of 10^−2^ or co-infected with 100 μL of active (a) or inactive (i) IAV DIPs DI244 or OP7. The DIP material used was diluted to 1:20. Cells were lysed at indicated time points for subsequent intracellular RNA isolation. Gene expression was quantified using real-time RT-qPCR and expressed as fold change (relative to untreated, uninfected cells (mock infection control)). The figure depicts the results of three independent experiments. Error bars indicate the SD. Upper panel: Two-way ANOVA (or mixed-effects model for IFN-λ-1) followed by Dunnett´s multiple comparison test (*** *p* < 0.001; ** *p* < 0.01; * *p* < 0.05; not significant, *p* > 0.05) was used to determine statistical significance compared to the untreated group. Lower panel: Two-way ANOVA followed by Tukey´s multiple comparison test was used to determine statistical significance between respective active and inactive DIP material.

We next studied host cell gene expression during inhibition of YFV replication by IAV DIP co-infection in A549 cells to identify mechanism of protection. For this, we investigated the expression of type-I and -III IFNs (IFN-β-1 and IFN-λ-1, respectively) and the ISGs Mx1 and RSAD2 using real-time RT-qPCR quantification (Figure 2). Co-treatment with active DIPs induced an increased expression of all genes at early time points (6 hpi, 24 hpi) relative to YFV infection alone (Figure 2, upper panel). The early upregulation of gene expression was independent on the infection with YFV, as infection with only IAV DIPs showed similar dynamics and levels (Figure 2, lower panel). A much less pronounced early upregulation of antiviral innate immunity was observed for infection with inactive DIPs, in agreement with the absence of inhibition by inactive DIPs (Figure 1A). For comparison, YFV infection without IAV DIPs resulted in relatively low IFN and ISG gene expression levels at early times. This suggests that the antiviral activity of IAV DIPs against YFV infection is caused by the early and strong upregulation of the type-I and -III IFN responses.

**Figure 3:**
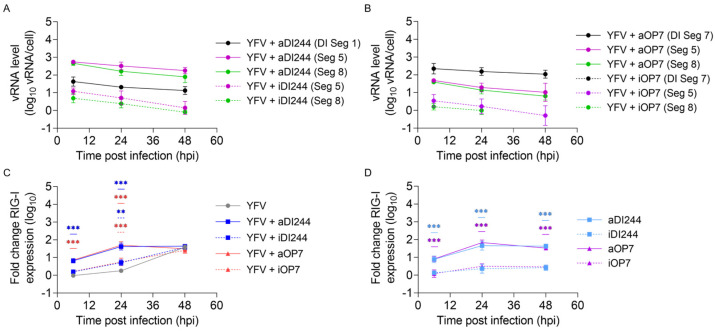
Intracellular levels of DI and FL vRNA and RIG-I expression after IAV DIP infections. A549 cells were infected with YFV alone at a MOI of 10^−2^ or co-infected with 100 μL of active (a) or inactive (i) IAV DIPs DI244 (**A**) or OP7 (**B**). The DIP material used for infection was diluted to 1:20. Cells were lysed at indicated time points for subsequent intracellular RNA isolation. (**A**,**B**) DI and FL vRNA levels were quantified using real-time RT-qPCR. (**C**,**D**) Gene expression of RIG-I was quantified using real-time RT-qPCR and expressed as fold change (compared to untreated, uninfected cells (mock infection control)). The figure depicts the results of three independent experiments. Error bars indicate the SD. (**C**) Two-way ANOVA followed by Dunnett´s multiple comparison test (*** *p* < 0.001; ** *p* < 0.01; * *p* < 0.05; not significant, *p* > 0.05) was used to determine statistical significance compared to the untreated group. (**D**) Two-way ANOVA followed by Tukey´s multiple comparison test was used to determine statistical significance between respective active and inactive DIP material.

Next, we measured the intracellular IAV vRNA levels of the respective DI vRNA and of FL vRNA of Seg 5 and 8 in co-infections with DI244 and OP7 (Figure 3). Moreover, we measured the gene expression of RIG-I, a host-derived PRR that detects vRNA and has a crucial role in initiating a cellular innate immune response. We found high levels of DI and FL vRNAs after co-infection (Figure 3A,B) and a concurrent early upregulation of RIG-I expression (Figure 3C). At 24 hpi, we detected an increase by factors of 29 (YFV + aOP7) and 24 (YFV + aDI244) in the expression of RIG-I for IAV DIP co-infections compared to YFV infection alone. This may explain the upregulation of type-I and -III IFN response relative to the infection without IAV DIPs (as shown in Figure 2). The same upregulation of RIG-I was observed after infection with only IAV DIPs (Figure 3D). Moreover, lower vRNA levels were observed for co-infections with inactive DIPs, and no DI vRNAs could be detected for inactive DI244 co-infections (Figure 3A,B). This indicates an efficient degradation of vRNAs by UV inactivation. Accordingly, lower RIG-I expression levels were observed upon inactive IAV DIP infections (Figure 3C,D), which may explain their lack of IFN-induced antiviral activity (Figure 1A). Furthermore, we detected no increase of IAV genomic FL and DI vRNAs over time, indicating the defect in virus replication of IAV DIPs.

Taken together, IAV DIP co-infections resulted in an inhibition of YFV replication, which was dependent on the production of IFNs. Furthermore, we found that IAV DIP infection enhanced the type-I and -III IFN response, which appears to be initiated by sensing of DI and FL IAV vRNAs by RIG-I. The early and enhanced cellular innate immune response by IAV DIP infection likely explains their antiviral effect against YFV infection.

### 3.2. IAV DIP Infection Inhibits ZIKV Replication via JAK/STAT Signaling

**Figure 4:**
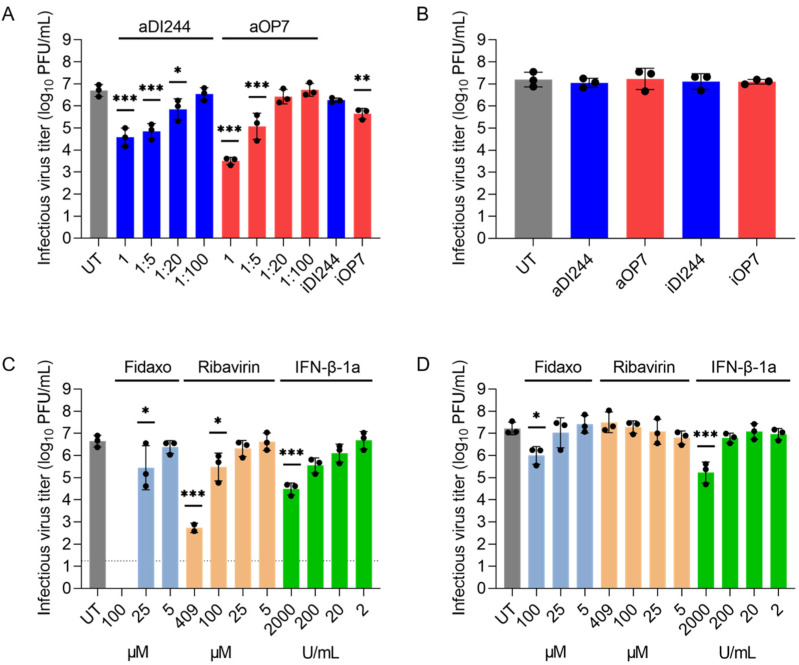
Inhibition of ZIKV replication and spread by IAV DIP co-infection in comparison to small molecule antiviral or IFN treatment. Infections in IFN-competent A549 cells (**A**,**C**) and in IFN-deficient Vero cells (**B**,**D**). Cells were infected with ZIKV alone at a MOI of 10^−2^ (UT, untreated), co-infected with 100 μL of active (a) or inactive (i) IAV DIPs DI244 or OP7 at indicated dilutions (**A**) or without dilution (**B**). In addition, ZIKV-infected cells were co-treated with antiviral drugs (**C**,**D**) at indicated concentrations. Infectious virus release is indicated by the ZIKV plaque titer (PFU/mL) at 72 hpi. The figure depicts the results of three independent experiments or two experiments for ribavirin (409 μM) in (**C**). Dashed horizontal line shows the LOD. Error bars indicate the SD. One-way ANOVA followed by Dunnett´s multiple comparison test (*** *p* < 0.001; ** *p* < 0.01; * *p* < 0.05; not significant, *p* > 0.05) was used to determine statistical significance compared to the untreated control group.

Next, we investigated the inhibition of ZIKV replication by IAV DIPs (Figure 4). Therefore, we infected A549 cells and Vero cells at a MOI of 10^−2^ with ZIKV alone (UT) or co-infected with IAV DIPs. For active DIPs (aDI244, aOP7), different dilutions ranging from undiluted (1) to 1:100 dilutions were tested. Here, active DIPs showed antiviral activity against ZIKV propagation in a dose-dependent manner (Figure 4A). For instance, undiluted material of active OP7 and DI244 reduced the infectious virus titer by more than three and two orders of magnitude, respectively. The antiviral activity of IAV DIPs against ZIKV replication appeared to be lower relative to the inhibition of YFV replication (Figure 1A). This may be due to the use of a live attenuated strain YFV-17D and its increased sensitivity to IFN treatment [79]. Moreover, we found a residual antiviral activity of inactive DIPs against ZIKV replication (Figure 4A). This is likely mediated by a weak stimulation of the IFN-induced antiviral gene expression upon infection with inactive IAV DIPs only (also see Figure 2).

In Vero cells, co-treatment with IAV DIPs did not result in an inhibition of ZIKV replication, indicating the dependence of the antiviral effect on the production of IFN (Figure 4B). Next, we tested different relevant antivirals (i.e., fidaxomicin (Fidaxo) [13], ribavirin [14,80], and IFN-β-1a [81,82]) against ZIKV infection (Figure 4C,D). As expected, the different drugs conferred an antiviral activity in a dose-dependent manner. In A549 cells, fidaxomicin at 100 μM showed very high inhibitory activity as no infectious virus titer could be detected anymore (Figure 4C). In comparison, a concentration of 409 μM of ribavirin and 2000 U/mL of IFN-β-1a reduced the infectious virus titer by almost four and more than two orders of magnitude, respectively. The inhibition conferred by the three different antivirals were less pronounced in Vero relative to A549 cells, indicating a lower sensitivity to treatment of the former (Figure 4D). Although Vero cells are unable to produce IFN [74,76], we found a residual inhibition of ZIKV replication during IFN-β-1a treatment (Figure 4D). This may be explained by the fact that Vero cells bear functional IFN receptors and can, thus, respond to exogenous IFN treatment [83,84].

**Figure 5:**
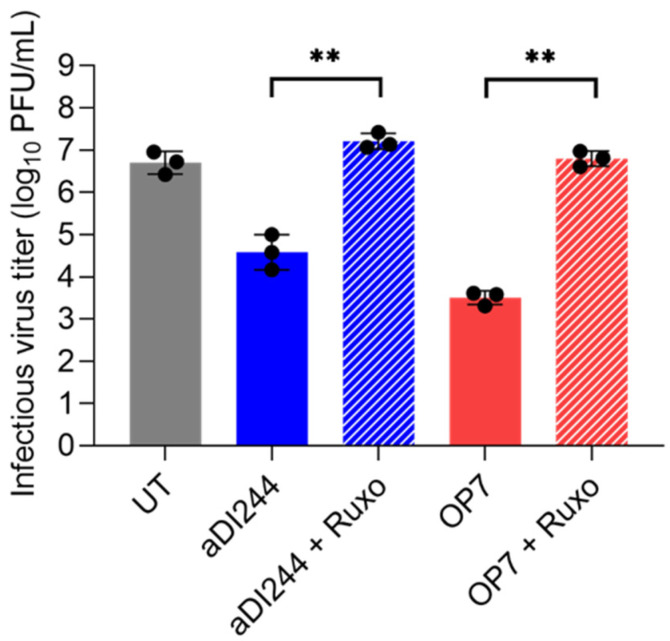
Antiviral activity of IAV DIPs against ZIKV propagation under JAK1/2 inhibition. A549 cells were infected with ZIKV alone at a MOI of 10^−2^ (UT, untreated) or co-infected with 100 μL of active (a) IAV DIPs DI244 or OP7. The DIP material used was non-diluted. As indicated, co-infections were performed in the presence of ruxolitinib (2 μM). Infectious virus release is indicated by the ZIKV plaque titer (PFU/mL) at 72 hpi. The figure depicts the results of three independent experiments. Error bars indicate the SD. One-way ANOVA followed by Tukey’s multiple comparison test (*** *p* < 0.001; ** *p* < 0.01; * *p* < 0.05; not significant, *p* > 0.05) was used to determine statistical significance between co-infections with and without ruxolitinib.

To confirm that the inhibition of ZIKV replication is caused by the ability of DIPs to stimulate the IFN system, we used the Janus kinase 1/2 (JAK1/2) inhibitor ruxolitinib in co-infection experiments (Figure 5). JAK1/2 is a crucial player in the JAK/STAT signaling pathway. Following binding of type-I IFNs to the IFN-α/β receptor, the associated JAK would stimulate activation of STAT, ultimately leading to expression of antiviral ISGs. For co-treatment with ruxolitinib, we observed no inhibition of infectious ZIKV release by IAV DIPs in A549 cells. In conclusion, the inhibition of ZIKV by IAV DIPs was dependent on IFN production and on signaling through the JAK/STAT pathway.

### 3.3. IFN-Dependent Inhibition of RSV Replication by IAV DIPs Relies on JAK/STAT Signaling

**Figure 6:**
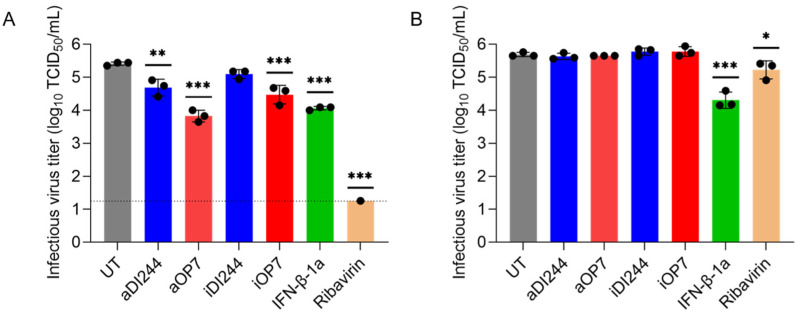
Inhibition of RSV replication and spread by IAV DIP co-infection in comparison to ribavirin or IFN treatment. Infections in IFN-competent A549 cells (**A**) or IFN-deficient Vero cells (**B**). Cells were infected with RSV alone at a MOI of 10^−2^ (UT, untreated), co-infected with 100 μL of active (a) or inactive (i) IAV DIPs DI244 or OP7. In addition, RSV-infected cells were co-treated with antiviral drugs (IFN-β-1a (2000 U/mL) or ribavirin (409 μM)). The DIP material used was diluted to 1:20. Infectious virus release is indicated by the RSV TCID_50_ titer (TCID_50_/mL) at 72 hpi. The figure depicts the results of three independent experiments. Dashed horizontal line shows the LOD. Error bars indicate the SD. One-way ANOVA followed by Dunnett´s multiple comparison test (*** *p* < 0.001; ** *p* < 0.01; * *p* < 0.05; not significant, *p* > 0.05) was used to determine statistical significance compared to the untreated group.

Finally, we tested the antiviral activity of IAV DIPs against RSV replication. For this, we infected A549 cells and Vero cells at a MOI of 10^−2^ (UT) or co-infected with IAV DIPs (diluted to 1:20) (Figure 6). RSV-infected A549 cells showed an infectious virus titer of 2.6 × 10^5^ TCID_50_/mL at 72 hpi. Co-treatment with active DI244 (aDI244) and active OP7 (aOP7) suppressed the infectious virus release to 5.4 × 10^4^ and 7.1 × 10^3^ TCID_50_/mL, respectively (Figure 6A). Furthermore, two clinically relevant antivirals, i.e., IFN-β-1a (2000 U/mL) and ribavirin (409 μM), were tested for comparison. Treatment with IFN-β-1a showed an inhibition of infectious virus release to 1.2 × 10^4^ TCID_50_/mL, whereas ribavirin treatment almost shut down RSV propagation (6 × 10^0^ TCID_50_/mL) in A549 cells.

**Figure 7:**
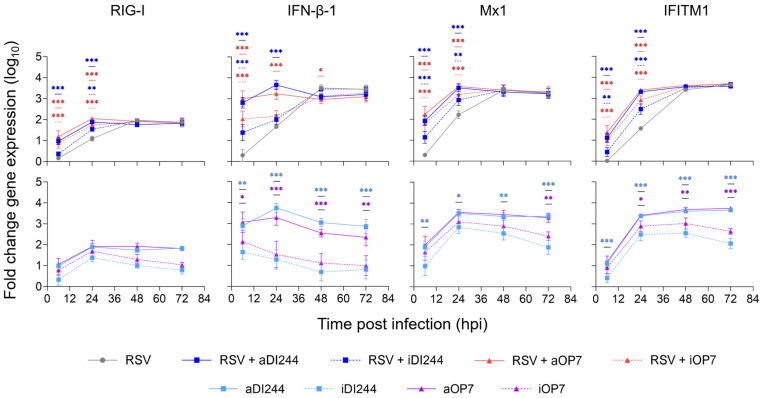
Induction of IFN-induced antiviral gene expression by IAV DIP infections. A549 cells were infected with RSV alone at a MOI of 10^−2^ or co-infected with 100 μL of active (a) or inactive (i) IAV DIPs DI244 or OP7. The DIP material used was diluted to 1:20. Cells were lysed at indicated time points for subsequent intracellular RNA isolation. Gene expression was quantified using real-time RT-qPCR and expressed as fold change (relative to untreated, uninfected cells (mock infection control)). The figure depicts the results of three independent experiments. Error bars indicate the SD. Upper panel: Two-way ANOVA followed by Dunnett´s multiple comparison test (*** *p* < 0.001; ** *p* < 0.01; * *p* < 0.05; not significant, *p* > 0.05) was used to determine statistical significance compared to the untreated group. Lower panel: Two-way ANOVA followed by Tukey’s multiple comparison test was used to determine statistical significance between respective active and inactive DIP material.

An early and enhanced upregulation of IFN-induced antiviral gene expression (indicated by RIG-I, IFN-β-1, Mx1, and IFITM1) was observed in A549 cells for the treatment with DIPs only and the co-infection with RSV in comparison to infection with RSV only (Figure 7). The residual antiviral activity of inactive DIPs against RSV infection (Figure 6A) may be explained by the intermediate upregulation of IFN and ISGs (Figure 7).

Next, we tested the inhibitory activity of IAV DIPs in Vero cells (Figure 6B). As expected, no suppression of the infectious virus titer was found for active or inactive IAV DIPs. However, treatment with IFN-β-1a led to a reduction in infectious virus titers from 4.9 × 10^5^ TCID_50_/mL (UT) to 2.3 × 10^4^ TCID_50_/mL, as Vero cells still express an IFN receptor. Further, a lower inhibition (compared to A549 cells, Figure 6A) was observed for ribavirin treatment (reduction to 1.9 × 10^5^ TCID_50_/mL).

**Figure 8:**
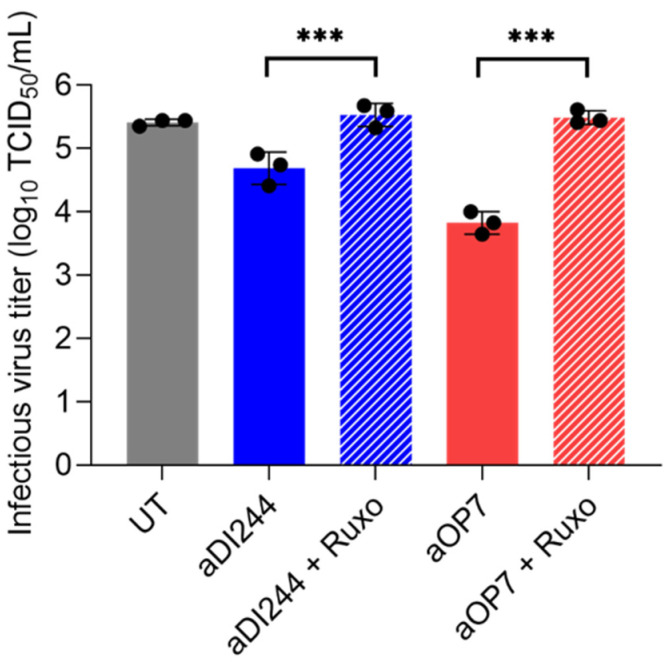
Antiviral activity of IAV DIPs against RSV propagation under JAK1/2 inhibition. A549 cells were infected with RSV alone at a MOI of 10^−2^ (UT, untreated) or co-infected with 100 μL of active (a) IAV DIPs DI244 or OP7. The DIP material used was diluted to 1:20. As indicated, co-infections were performed in the presence of ruxolitinib (2 µM, after pre-treatment for 3 h). Infectious virus release is indicated by the RSV TCID_50_ titer (TCID_50_/mL) at 72 hpi. The figure depicts the results of three independent experiments. Error bars indicate the SD. One-way ANOVA followed by Tukey´s multiple comparison test (*** *p* < 0.001; ** *p* < 0.01; * *p* < 0.05; not significant, *p* > 0.05) was used to determine statistical significance between co-infections with and without ruxolitinib.

To investigate whether the inhibition of RSV infection by active IAV DIPs is dependent on signaling through the JAK/STAT pathway, A549 cells were co-infected with aDI244 or aOP7 in the presence or absence of the JAK1/2 inhibitor ruxolitinib (Ruxo) (Figure 8). No suppression of the infectious virus titer was found in presence of ruxolitinib. Taken together, we show that IAV DIPs inhibit RSV propagation in an IFN-dependent manner. Moreover, the antiviral activity was promoted via signaling through the JAK/STAT pathway, which results in IFN-induced antiviral gene expression.

## 4. Discussion

IAV DIPs are considered for use as broad-spectrum antiviral agents to treat not only infections of different IAV strains [39,41,42,52,53] but also those of unrelated viruses [22,23,24]. Our study shows that IAV DIPs also inhibit the replication of IFN-sensitive RSV, YFV, and ZIKV infections in vitro by their ability to upregulate the IFN response that acts antivirally. The availability of such broadly acting antivirals may increase our pandemic preparedness.

Our study supports other work that suggests that IFN induction is a key player for the antiviral activity of DIPs against the replication of unrelated viruses [22,23,24,25,33,42,54,55]. IAV DIPs showed a lower antiviral activity against ZIKV than for YFV replication. In our study, we used YFV-17D, a live-attenuated vaccine strain, and wild-type (WT) ZIKV. Previously, it has been shown that in type-I IFN-deficient mice, type-II IFN restricted the replication of YFV-17D, but not that of WT YFV [79], suggesting that YFV-17D is more sensitive to IFN treatment than WT YFV. With respect to the present study, this may explain the higher antiviral activity of IAV DIPs against YFV-17D compared to antiviral activity against WT ZIKV replication, a phylogenetically related virus. Accordingly, other viruses that demonstrate higher IFN antagonism ability (like the more virulent WT YFV [79]) should be less susceptible to inhibition by IAV DIPs.

DI and FL vRNAs of IAV DIPs are potent RIG-I ligands [43,57], which is a cytosolic PRR that can initiate an IFN response. In line with this, we observed an upregulation of RIG-I upon infection with IAV DIPs, consistent with previous reports [22]. We also tested infections with IAV DIPs that were inactivated by UV light, which results in photodimeric lesions [85] or unspecific chain breaks [86,87] of their vRNAs. Here, we observed no or a very weak antiviral effect, likely due to the reduced activation of RIG-I. Accordingly, the residual antiviral activity of inactive IAV DIPs against ZIKV replication may be explained by the weak stimulation of the IFN response observed in our studies.

Next, we showed that the antiviral effect against ZIKV and RSV was dependent on an IFN-induced innate immune response that involved the JAK/STAT pathway, confirming previous IAV DIP co-infection studies with SARS-CoV-2 [22]. Furthermore, the ISGs Mx1, RSAD2, and IFITM1 were upregulated early after infection with IAV DIPs compared to infections with YFV or RSV only. This early stimulation of an antiviral state likely explains the antiviral effect of IAV DIPs against unrelated viruses, as suggested previously [22], and also for DENV DIPs [25].

In agreement with this body of evidence, we observed an absence of antiviral activity against RSV, YFV, and ZIKV in Vero cells, which are deficient in IFN production [74,76] due to a deleted region in chromosome 12 encoding for numerous type-I IFN genes [78]. Previously, Easton et al. showed that mice were protected from death after administration of DI244 and an otherwise lethal dose of pneumonia virus of mice. In type-I IFN deficient mice, only 17% survived, but a delayed onset of symptoms was observed [23]. Moreover, a reduced protection in type-I IFN-deficient mice was found against influenza B virus infection [24]. Nevertheless, yet unidentified pathways may also be involved for IAV DIPs inducing an antiviral state in an IFN-independent manner [23], as also speculated by others [88,89].

The inhibition of infectious RSV and ZIKV release by IAV DIPs was comparable to IFN-β-1a treatment. With respect to clinical studies, partial positive effects of type-I IFN treatment on RSV infection were observed [90,91], but other studies resulted in no effects [92,93]. For RSV A2 infections, little inhibition by IFN treatment was observed [94]. Inhibition of ZIKV replication by exogenous IFN-β and -λ-1 treatment in human vaginal and cervical epithelial cells was modest [17]. Disadvantages involved in the therapy with recombinant type-I IFN are the high costs [18] and the risk of adverse effects such as flu-like symptoms, fatigue, depression, neutropenia, and anemia [19,20,21]. Compared to treatment with recombinant IFN, it is suggested that IAV DIPs can stimulate a more physiological IFN response in target tissues [22]. In line with this, the administration of IAV DIPs is typically very well tolerated in mice, resulting in no apparent adverse or toxic effects [38,39,60]. Furthermore, high-yield, cell culture-based production and purification of IAV DIPs is feasible [38,60,95]. This suggests that IAV DIPs can be produced in an economical manner, and thus, we anticipate relatively low prices per dose.

In our experiments, we observed that the small molecule antivirals ribavirin (in ZIKV and RSV infection) and fidaxomicin (ZIKV infection) conferred a higher antiviral activity than IAV DIPs. However, ribavirin approved for treatment of RSV infection in humans is associated with high costs and possible toxic effects [8,10]. Moreover, fidaxomicin and ribavirin are not approved for treatment of humans infected with ZIKV. Next, we showed that ribavirin and fidaxomicin treatment resulted in higher antiviral effects in A549 cells than in Vero cells. For ZIKV infection, no antiviral effect was observed for ribavirin treatment of Vero cells. These results suggest that Vero cells are less sensitive to antiviral treatment, as already reported for ribavirin for suppression of ZIKV replication [13]. This cell-dependent antiviral activity underlines the importance of the selected testing system concerning the antiviral effect observed in vitro. However, this may not necessarily represent the degree of inhibition in vivo and thus may not allow for a relevant comparison of different antivirals considered for human use. Therefore, it is mandatory to perform additional studies to compare the tested antiviral agents, including the IAV DIPs, in animal models and carefully designed clinical trials. In this manner, the antiviral activity and side effects at different doses with different routes and times of administration can be investigated to draw conclusions about their comparability and applicability.

The main application of IAV DIPs could be the intranasal application to treat IFN-sensitive respiratory virus infections, including that of IAV [39,96], SARS-CoV-2 [22] and RSV (this study). For instance, in mice and ferrets, this route of administration resulted in an antiviral effect against IAV [38,39,53,60]. The inhibition of YFV and ZIKV as shown in the present study may be regarded as a result from an in vitro model that indicates that IAV DIPs also exhibit antiviral activity against the replication of other IFN-sensitive viruses. The optimal route of administration of IAV DIPs and antiviral efficacy in animal experiments against such systemic viral infections, however, remain to be elucidated.

In the future, the availability of broadly acting antivirals like IAV DIPs could allow for a rapid countermeasure to protect susceptible people and persons at risk and help to contain the spread of newly emerging IFN-sensitive viruses in case of a pandemic, when vaccines or other antivirals are not available yet.

## 5. Patents

A patent for the use of OP7 as antiviral agent for treatment of IAV infection is approved for USA and pending for European Union and Japan. Patent holders are S.Y.K. and U.R. Another patent for the use of DI244 and OP7 as an antiviral agent for treatment of coronavirus infection is pending. Patent holders are S.Y.K., U.R., and M.D.H.

## Figures and Tables

**Table 1:** IAV DIP material used for co-infection experiments.

Description	HA Titer ^1^ (log_10_ HAU/100 µL)	DI vRNA Concentration (DI vRNAs/mL) ^2^
Active OP7 (8 min UV)	3.53	1.60 × 10^11^
Inactive OP7 (24 min UV)	3.52	2.85 × 10^10^
Active DI244 (no UV)	3.89	8.89 × 10^10^
Inactive DI244 (24 min UV)	3.85	1.03 × 10^9^

^1^ Hemagglutination assay (HA) [63]. ^2^ OP7: Seg 7-OP7 vRNAs/mL, DI244: Seg 1-DI244 vRNAs/mL.

**Table 2:** Primers used for real-time RT-qPCR (gene expression).

Target Gene	Primer Name	Sequence (5′→3′)
RIG-I	RIG-I for	GGACGTGGCAAAACAAATCAG
[68]	RIG-I rev	GCAATGTCAATGCCTTCATCA
IFN-β-1	IFN-β-1 for	CATTACCTGAAGGCCAAGGA
[69]	IFN-β-1 rev	CAGCATCTGCTGGTTGAAGA
IFN-λ-1	IFN-λ-1 for	GGTGACTTTGGTGCTAGGCT
[69]	IFN-λ-1 rev	TGAGTGACTCTTCCAAGGCG
Mx1	Mx1 for	GTATCACAGAGCTGTTCTCCTG
[69]	Mx1 rev	CTCCCACTCCCTGAAATCTG
IFITM1	IFITM1 for	ATCAACATCCACAGCGAGAC
[70]	IFITM1 rev	CAGAGCCGAATACCAGTAACAG
RSAD2	RSAD2 for	CCCCAACCAGCGTCAACTAT
[69]	RSAD2 rev	TGATCTTCTCCATACCAGCTTCC
GAPDH	GAPDH for	CTGGCGTCTTCACCACCATGG
[69]	GAPDH rev	CATCACGCCACAGTTTCCCGG

RIG-I: retinoic acid-inducible gene-I.

## Data Availability

The data presented in this study are available in Supplemental Table S1.

## Supplementary Materials

The following supporting information can be downloaded at: https://www.mdpi.com/article/10.3390/v15091872/s1.
